# Di-μ_2_-acetato-diacetato-bis­{μ_2_-3,3′,5,5′-tetra­meth­oxy-2,2-[ethane-1,2-diylbis(nitrilo­methyl­idyne)]diphenolato}tricobalt(II,III) dichloro­methane disolvate

**DOI:** 10.1107/S1600536811003783

**Published:** 2011-02-05

**Authors:** Gervas E. Assey, Ray J. Butcher, Yilma Gultneh

**Affiliations:** a Department of Chemistry, Howard University, 525 College Street NW, Washington, DC 20059, USA

## Abstract

The trinuclear title compound, [Co_3_(CH_3_COO)_4_(C_20_H_22_N_2_O_6_)_2_]·2CH_2_Cl_2_, contains mixed-valence cobalt ions in the following order Co^III^–Co^II^–Co^III^ where all the three cobalt ions are hexa­coordinated. The central cobalt ion is situated on an inversion centre and is in an all-oxygen environment, coordinated by four phenolate O atoms and two O atoms from bridging acetate groups, while the terminal cobalt ion is hexa­coordinated by two phenolate O atoms, two acetate O atoms and two imine N atoms. This complex contains a high-spin central Co^II^ and two terminal low-spin Co^III^ *i.e.* Co^III^(S = 0)–Co^II^(S = 3/2)-Co^III^(S = 0). There are weak inter­molecular C—H⋯O inter­actions involving the meth­oxy groups, as well as inter­molecular C—H⋯O inter­actions involving the acetate anions. In addition, the dichoromethane solvate mol­ecules are held in place by weak C—H⋯Cl inter­actions.

## Related literature

For background to to the use of transition metal complexes with Schiff bases as potential enzyme inhibitors, see: You *et al.* (2008 ▶); Shi *et al.* (2007 ▶). For the use of transition metal complexes for the development of catalysis, magnetism and mol­ecular architectures, see: Yu *et al.* (2007 ▶); You & Zhu (2004 ▶); You & Zhou (2007 ▶). For the use of transition metal complexes for optoelectronic and also for photo- and electro­luminescence applications, see: Yu *et al.* (2008 ▶). For the potential use of transition metal complexes in the modeling of multisite metalloproteins and in nano-science, see: Chattopadhyay *et al.* (2006 ▶). For the importance of tri-nuclear cobalt Schiff base complexes as catalysts for organic mol­ecules and as anti­viral agents due to their ability to inter­act with proteins and nucleic acids, see: Chattopadhyay *et al.* (2006 ▶, 2008 ▶); Babushkin & Talsi (1998) ▶. For background to metallosalen complexes, see: Dong *et al.* (2008 ▶). For the magnetic properties of quadridentate metal complexes of Schiff bases, see: He *et al.* (2006 ▶); Gerli *et al.* (1991 ▶). For the anti­microbial activity of Schiff base ligands and their complexes, see: You *et al.* (2004 ▶). 
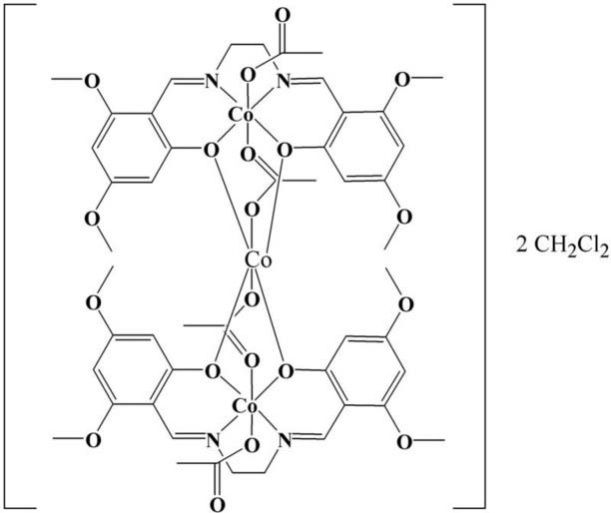


## Experimental

### 

#### Crystal data

[Co_3_(C_2_H_3_O_2_)_4_(C_20_H_22_N_2_O_6_)_2_]·2CH_2_Cl_2_
*M* *_r_* = 1355.61
Monoclinic, 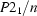

*a* = 13.9235 (9) Å
*b* = 13.4407 (8) Å
*c* = 16.0019 (11) Å
β = 112.724 (8)°
*V* = 2762.2 (3) Å^3^
*Z* = 2
Cu *K*α radiation
μ = 9.45 mm^−1^
*T* = 110 K
0.42 × 0.25 × 0.18 mm

#### Data collection

Oxford Diffraction Xcalibur diffractometer with a Ruby detector
Absorption correction: multi-scan (*CrysAlis PRO*; Oxford Diffraction, 2009 ▶) *T*_min_ = 0.320, *T*_max_ = 1.000
10708 measured reflections
5306 independent reflections
3777 reflections with *I* > 2σ(*I*)
*R*_int_ = 0.043

#### Refinement

*R*[*F* ^2^ > 2σ(*F* ^2^)] = 0.083
*wR*(*F* ^2^) = 0.251
*S* = 1.03
5306 reflections
373 parameters
H-atom parameters constrained
Δρ_max_ = 1.11 e Å^−3^
Δρ_min_ = −1.66 e Å^−3^

### 

Data collection: *CrysAlis PRO* (Oxford Diffraction, 2009 ▶); cell refinement: *CrysAlis PRO*; data reduction: *CrysAlis PRO*); program(s) used to solve structure: *SHELXS97* (Sheldrick, 2008 ▶); program(s) used to refine structure: *SHELXL97* (Sheldrick, 2008 ▶); molecular graphics: *SHELXTL* (Sheldrick, 2008 ▶); software used to prepare material for publication: *SHELXTL*.

## Supplementary Material

Crystal structure: contains datablocks I, global. DOI: 10.1107/S1600536811003783/jj2072sup1.cif

Structure factors: contains datablocks I. DOI: 10.1107/S1600536811003783/jj2072Isup2.hkl

Additional supplementary materials:  crystallographic information; 3D view; checkCIF report

## Figures and Tables

**Table 1:** Hydrogen-bond geometry (Å, °)

*D*—H⋯*A*	*D*—H	H⋯*A*	*D*⋯*A*	*D*—H⋯*A*
C—H0*A*⋯O22*A*	0.99	2.33	3.269 (13)	158
C4—H4*A*⋯O6^i^	0.98	2.35	3.326 (8)	175
C7—H7*A*⋯O6^ii^	0.98	2.51	3.421 (9)	156
C11—H11*A*⋯O3^ii^	0.99	2.62	3.602 (8)	174
C11—H11*B*⋯Cl1^iii^	0.99	2.73	3.664 (8)	158
C15—H15*A*⋯O4^iv^	0.98	2.64	3.568 (10)	158
C12*A*—H12*B*⋯Cl1^ii^	0.98	2.91	3.354 (8)	108

Symmetry codes: (i) 
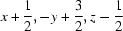
; (ii) 
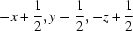
; (iii) 
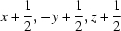
; (iv) 
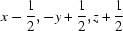
.

**Fig. 1.**
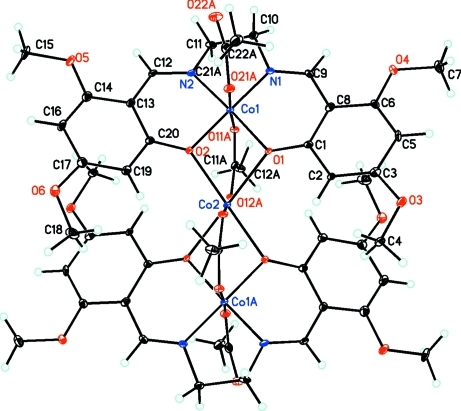
Diagram of trinuclear C48H56Co3N4O20 unit showing atom labeling. Thermal ellipsoids are at the 30% probability level.

**Fig. 2.**
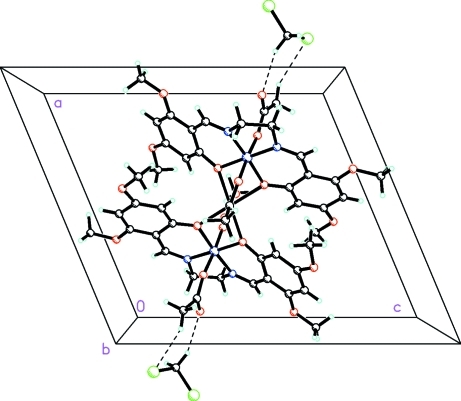
The molecular packing for C48H56Co3N4O20.2(CH2Cl2) viewed down the b axis. C—H···Cl and C—H···O interactions bonds are shown by dashed lines.

## supplementary crystallographic information

### Comment

A number of transition metal complexes with Schiff base ligands have been studied as potential inhibitors for the xanthine oxidase (XO) enzyme (You *et al.*, 2008) and also for the jack bean urease enzyme (jbU) (Shi *et al.*, 2007). The enzyme XO catalyzes the hydroxylation of hypoxanthine and xanthine to yield uric acid and superoxide anions. Other areas where complexes of transition metals have played roles are in the development of catalysis, magnetism and molecular architecture (Yu *et al.*, 2007, You & Zhu, 2004, You & Zhou, 2007). Complexes of transition metals with Schiff base ligands have also been shown to be useful materials for optoelectronics and also for photo and electro-luminance applications (Yu *et al.*, 2008). Studies for antimicrobial activities of Schiff base ligands as well as those of their corresponding complexes have been investigated (You *et al.*, 2004) where it was shown that Schiff base ligands as well as their complexes exhibited good antibacterial properties. Metallosalen complexes are of great importance due to their use in various catalytic chemical transformations that includes, epoxidation of olefins, symmetric ring opening, azirdination of olefins, olefine cyclopropanation and formation of linear and cyclic hydrocarbonation (Dong *et al.*, 2008).

The importance of tri-nuclear cobalt Schiff base complexes ranges from, catalysts for oxidation of organic molecules, antiviral agents due to their ability to interact with proteins and nucleic acids and they have also used to mimic the biological co-factor such as cobalamin (Chattopadhyay *et al.*, 2008, Babushkin & Talsi, 1998). The quadridentate metal complexes of Schiff bases have been studied extensively as B12 models, their magnetic interaction between bridged paramagnetic metal ions and their applications (Gerli *et al.*, 1991). Magnetic susceptibilities data for the trinuclear mixed valence compound [Co^II^(OAc)_2_(hapt)_2_Co_2_(III)(py)_2_](ClO_4_)_2_ [where (hapt) is bis-(2-hydroxyacetophenone) trimethylenediimine] were measured in the temperature range of 300–2 K and it was found that µ_eff_ values are almost constant ranging from 4.37 to 5.00 BM (He *et al.*, 2006). The values obtained suggested that the oxidation states are Co^III^(S = 0)-Co^II^(S = 3/2)-Co^III^(S = 0). Cyclic tri-nuclear cobalt complexes have also shown some catalytic activities in epoxidation of olefins, autoxidation of hydrocarbons, utility in modeling multinuclear active sites of metalloproteins and their potential use in nanoscience (Chattopadhyay *et al.*, 2006).

The title compound C_50_H_60_Cl_4_Co_3_N_4_O_20_ is a trinuclear cobalt Schiff base complex containing a central high spin Co^II^ and two terminal low spin Co^III^ centers. The environment around Co(1) is hexacoordinated with two imine nitrogen atoms, N(1) and N(2), two phenolate oxygen atoms, O(1) and O(2), and two oxygen atoms, O(11 A) and O(21 A), from two acetate groups. The central Co(2) ion is coordinated by four phenolate oxygen atoms and two acetate oxygen atoms O(12 A), O(2)#2, O(2), O(1), O(1), O(1)#1 and O(12)#1. The bond distances of the coordination atoms around Co(1) are Co(1)—N(2) = 1.861 (5) Å, Co(1)—N(1) = 1.871 (5) Å, Co(1)—O(2) = 1.887 (4) Å, Co(1)—O(1) = 1.89 (4) Å, Co(1)—O(21 A) = 1.891 (4) Å, Co(1)—O(11 A) = 1.929 (4) Å and the bond lengths between Co(2) and its coordinating atoms are Co(2)—O(12 A)#1 = 2.043 (4) Å, Co(2)—O(12 A) = 2.043 (4) Å, Co(2)—O(2)#1 = 2.117 (4) Å, Co(A)—O(2) = 2.117 (4) Å, Co(2)—O(1) = 2.160 (4) Å, Co(2)—O(1)#1 = 2.160 (4) Å. The coordination around the central metal ion displays a slight distortion from octahedral geometry as shown by the *cis* angles are mostly close to 90°. The main deviations are caused by the small bite of the salen O donors [72.15 (15)°]. The basal planes of the complex are formed by the two bridging O atoms and two N atoms of the Schiff base ligand. The O atoms of the acetate group occupy apical positions.

There are weak intermolecular C—H···O interactions involving the methoxy groups and acetate anions. In addition the dichoromethane solvate molecules are held in place by weak C—H···Cl interactions.

### Experimental

The synthesis of the ligand ethylene-bis(2,4-dimethoxy-salicylaldimine) was achieved by adding a solution of (2 g, 33.3 mmol) ethylenediamine in 25 ml s of methanol to the solution of (12.13 g, 66.6 mmol) 2,4-dimethoxysalicylaldehyde in 40 ml s of methanol. The mixture was refluxed overnight while stirring. The reaction mixture was then evaporated under reduced pressure to afford yellow solids.

The synthesis of the complex C_50_H_60_Cl_4_Co_3_N_4_O_20_ was accomplished by adding a solution of (0.38 g, 1 mmol) of ethylene-bis(2,4-dimethoxy-salicylaldimine) in 20 ml dichloromethane to a solution of Co(CH_3_COO)_2_.H_2_O in 5 ml me thanol. The mixture was stirred for 3 h, filtered and layered with di-ethyl ether for crystallization. Crystals suitable for X-ray diffraction were obtained.

### Refinement

H atoms were placed in geometrically idealized positions and constrained to ride on their parent atoms with a C—H distances of 0.95 and 0.99 Å *U*_iso_(H) = 1.2*U*_eq_(C) and 0.98 Å for CH_3_ [*U*_iso_(H) = 1.5*U*_eq_(C)]. In the final difference Fourier the maximum and minimum electron density of 1.11 and -1.66 e^-^/Å^3^ were located 0.93 Å and 0.44 Å from H0A and Cl1 respectively

### Crystal data

**Table:**

[Co_3_(C_2_H_3_O_2_)_4_(C_20_H_22_N_2_O_6_)_2_]·2CH_2_Cl_2_	*F*(000) = 1394
*M**_r_* = 1355.61	*D*_x_ = 1.630 Mg m^−^^3^
Monoclinic, *P*2_1_/*n*	Cu *K*α radiation, λ = 1.54178 Å
Hall symbol: -P 2yn	Cell parameters from 4463 reflections
*a* = 13.9235 (9) Å	θ = 4.4–73.9°
*b* = 13.4407 (8) Å	µ = 9.45 mm^−^^1^
*c* = 16.0019 (11) Å	*T* = 110 K
β = 112.724 (8)°	Thick needle, red-brown
*V* = 2762.2 (3) Å^3^	0.42 × 0.25 × 0.18 mm
*Z* = 2	

### Data collection

**Table:**

Oxford Diffraction Xcalibur diffractometer with a Ruby (Gemini Cu) detector	5306 independent reflections
Radiation source: Enhance (Cu) X-ray Source	3777 reflections with *I* > 2σ(*I*)
graphite	*R*_int_ = 0.043
Detector resolution: 10.5081 pixels mm^-1^	θ_max_ = 74.2°, θ_min_ = 4.5°
ω scans	*h* = −17→13
Absorption correction: multi-scan (*CrysAlis PRO*; Oxford Diffraction, 2009)	*k* = −16→13
*T*_min_ = 0.320, *T*_max_ = 1.000	*l* = −19→18
10708 measured reflections	

### Refinement

**Table:**

Refinement on *F*^2^	Primary atom site location: structure-invariant direct methods
Least-squares matrix: full	Secondary atom site location: difference Fourier map
*R*[*F*^2^ > 2σ(*F*^2^)] = 0.083	Hydrogen site location: inferred from neighbouring sites
*wR*(*F*^2^) = 0.251	H-atom parameters constrained
*S* = 1.03	*w* = 1/[σ^2^(*F*_o_^2^) + (0.1718*P*)^2^ + 2.5393*P*] where *P* = (*F*_o_^2^ + 2*F*_c_^2^)/3
5306 reflections	(Δ/σ)_max_ < 0.001
373 parameters	Δρ_max_ = 1.11 e Å^−^^3^
0 restraints	Δρ_min_ = −1.66 e Å^−^^3^

### Special details

**Table:**

Geometry. All e.s.d.'s (except the e.s.d. in the dihedral angle between two l.s. planes) are estimated using the full covariance matrix. The cell e.s.d.'s are taken into account individually in the estimation of e.s.d.'s in distances, angles and torsion angles; correlations between e.s.d.'s in cell parameters are only used when they are defined by crystal symmetry. An approximate (isotropic) treatment of cell e.s.d.'s is used for estimating e.s.d.'s involving l.s. planes.
Refinement. Refinement of *F*^2^ against ALL reflections. The weighted *R*-factor *wR* and goodness of fit *S* are based on *F*^2^, conventional *R*-factors *R* are based on *F*, with *F* set to zero for negative *F*^2^. The threshold expression of *F*^2^ > σ(*F*^2^) is used only for calculating *R*-factors(gt) *etc*. and is not relevant to the choice of reflections for refinement. *R*-factors based on *F*^2^ are statistically about twice as large as those based on *F*, and *R*- factors based on ALL data will be even larger.

### Fractional atomic coordinates and isotropic or equivalent isotropic displacement parameters (Å^2^)

**Table:**

	*x*	*y*	*z*	*U*_iso_*/*U*_eq_	
Co1	0.31088 (7)	0.37441 (7)	0.38337 (6)	0.0133 (3)	
Co2	0.5000	0.5000	0.5000	0.0138 (3)	
Cl1	−0.1730 (2)	0.4911 (2)	0.0248 (2)	0.0736 (8)	
Cl2	−0.2861 (3)	0.3805 (3)	0.1142 (2)	0.0826 (10)	
O1	0.4170 (3)	0.4463 (3)	0.3637 (3)	0.0142 (8)	
O2	0.3510 (3)	0.4519 (3)	0.4897 (3)	0.0176 (9)	
O3	0.5670 (4)	0.6103 (3)	0.1809 (3)	0.0229 (10)	
O4	0.3576 (4)	0.3258 (4)	0.0695 (3)	0.0239 (10)	
O5	0.0593 (4)	0.3797 (4)	0.5633 (3)	0.0276 (11)	
O6	0.2587 (4)	0.6707 (4)	0.6799 (3)	0.0279 (11)	
O11A	0.4076 (3)	0.2697 (3)	0.4437 (3)	0.0178 (9)	
O12A	0.5482 (3)	0.3568 (3)	0.5344 (3)	0.0182 (9)	
O21A	0.2186 (3)	0.4771 (3)	0.3178 (3)	0.0213 (10)	
O22A	0.0637 (4)	0.3991 (4)	0.2533 (3)	0.0296 (11)	
N1	0.2737 (4)	0.2957 (4)	0.2792 (3)	0.0154 (10)	
N2	0.2130 (4)	0.3016 (4)	0.4107 (3)	0.0179 (11)	
C	−0.1698 (10)	0.3882 (8)	0.0942 (7)	0.062 (3)	
H0A	−0.1096	0.3946	0.1527	0.075*	
H0B	−0.1608	0.3263	0.0644	0.075*	
C1	0.4274 (4)	0.4539 (5)	0.2860 (4)	0.0146 (12)	
C2	0.4903 (4)	0.5312 (5)	0.2754 (4)	0.0129 (11)	
H2A	0.5195	0.5790	0.3222	0.015*	
C3	0.5093 (5)	0.5373 (5)	0.1979 (4)	0.0190 (13)	
C4	0.6125 (5)	0.6836 (5)	0.2508 (4)	0.0265 (15)	
H4A	0.6550	0.7298	0.2323	0.040*	
H4B	0.6563	0.6503	0.3072	0.040*	
H4C	0.5570	0.7205	0.2607	0.040*	
C5	0.4666 (5)	0.4690 (5)	0.1259 (4)	0.0188 (13)	
H5A	0.4817	0.4740	0.0731	0.023*	
C6	0.4019 (5)	0.3940 (5)	0.1342 (4)	0.0170 (12)	
C7	0.3851 (7)	0.3271 (6)	−0.0085 (5)	0.0353 (18)	
H7A	0.3580	0.2671	−0.0448	0.053*	
H7B	0.4611	0.3288	0.0114	0.053*	
H7C	0.3550	0.3861	−0.0452	0.053*	
C8	0.3786 (5)	0.3854 (5)	0.2132 (4)	0.0179 (12)	
C9	0.3083 (4)	0.3071 (5)	0.2162 (4)	0.0150 (12)	
H9A	0.2861	0.2607	0.1678	0.018*	
C10	0.1969 (5)	0.2181 (5)	0.2716 (4)	0.0210 (13)	
H10A	0.2179	0.1549	0.2518	0.025*	
H10B	0.1280	0.2376	0.2261	0.025*	
C11	0.1902 (6)	0.2044 (5)	0.3629 (5)	0.0267 (15)	
H11A	0.1197	0.1814	0.3551	0.032*	
H11B	0.2413	0.1538	0.3987	0.032*	
C12	0.1734 (4)	0.3272 (5)	0.4676 (4)	0.0176 (12)	
H12A	0.1235	0.2839	0.4752	0.021*	
C13	0.1990 (5)	0.4164 (5)	0.5206 (4)	0.0181 (13)	
C14	0.1394 (5)	0.4440 (5)	0.5709 (4)	0.0238 (14)	
C15	−0.0028 (6)	0.4037 (6)	0.6139 (5)	0.0316 (17)	
H15A	−0.0562	0.3525	0.6038	0.047*	
H15B	−0.0363	0.4684	0.5941	0.047*	
H15C	0.0417	0.4067	0.6786	0.047*	
C16	0.1603 (5)	0.5288 (5)	0.6242 (5)	0.0231 (14)	
H16A	0.1192	0.5456	0.6577	0.028*	
C17	0.2433 (5)	0.5885 (5)	0.6274 (4)	0.0223 (14)	
C18	0.3486 (6)	0.7310 (6)	0.6947 (6)	0.041 (2)	
H18A	0.3498	0.7866	0.7347	0.061*	
H18B	0.3458	0.7570	0.6366	0.061*	
H18C	0.4116	0.6907	0.7229	0.061*	
C19	0.3059 (5)	0.5648 (5)	0.5799 (4)	0.0185 (13)	
H19A	0.3616	0.6072	0.5825	0.022*	
C20	0.2851 (4)	0.4775 (5)	0.5283 (4)	0.0178 (13)	
C11A	0.5005 (5)	0.2775 (5)	0.5022 (4)	0.0175 (12)	
C12A	0.5555 (5)	0.1810 (5)	0.5336 (5)	0.0272 (15)	
H12B	0.5189	0.1282	0.4910	0.041*	
H12C	0.5569	0.1651	0.5938	0.041*	
H12D	0.6270	0.1863	0.5367	0.041*	
C21A	0.1218 (5)	0.4706 (5)	0.2639 (4)	0.0210 (13)	
C22A	0.0821 (6)	0.5634 (6)	0.2093 (5)	0.0308 (16)	
H22A	0.0067	0.5580	0.1758	0.046*	
H22B	0.1163	0.5717	0.1665	0.046*	
H22C	0.0974	0.6210	0.2500	0.046*	

### Atomic displacement parameters (Å^2^)

**Table:**

	*U*^11^	*U*^22^	*U*^33^	*U*^12^	*U*^13^	*U*^23^
Co1	0.0088 (5)	0.0168 (5)	0.0142 (5)	−0.0035 (4)	0.0043 (3)	−0.0026 (4)
Co2	0.0088 (6)	0.0186 (7)	0.0142 (6)	−0.0039 (5)	0.0046 (5)	−0.0031 (5)
Cl1	0.0671 (18)	0.0709 (19)	0.0748 (18)	−0.0053 (15)	0.0184 (15)	0.0120 (15)
Cl2	0.087 (2)	0.093 (2)	0.075 (2)	−0.0023 (19)	0.0400 (18)	0.0040 (18)
O1	0.0090 (19)	0.020 (2)	0.0145 (19)	−0.0039 (17)	0.0058 (15)	−0.0011 (17)
O2	0.0118 (19)	0.024 (2)	0.019 (2)	−0.0044 (18)	0.0085 (16)	−0.0089 (18)
O3	0.023 (2)	0.022 (2)	0.027 (2)	−0.0050 (19)	0.0145 (19)	0.001 (2)
O4	0.025 (2)	0.033 (3)	0.014 (2)	−0.005 (2)	0.0088 (18)	−0.004 (2)
O5	0.017 (2)	0.035 (3)	0.037 (3)	−0.004 (2)	0.017 (2)	−0.005 (2)
O6	0.023 (2)	0.031 (3)	0.034 (3)	0.004 (2)	0.016 (2)	−0.010 (2)
O11A	0.013 (2)	0.019 (2)	0.018 (2)	−0.0029 (17)	0.0019 (16)	−0.0023 (17)
O12A	0.010 (2)	0.022 (2)	0.019 (2)	−0.0030 (17)	0.0019 (16)	−0.0040 (18)
O21A	0.014 (2)	0.023 (2)	0.024 (2)	0.0015 (18)	0.0034 (17)	−0.0020 (19)
O22A	0.023 (2)	0.024 (3)	0.034 (3)	−0.006 (2)	0.002 (2)	0.001 (2)
N1	0.011 (2)	0.017 (2)	0.016 (2)	−0.001 (2)	0.0028 (18)	−0.004 (2)
N2	0.012 (2)	0.020 (3)	0.021 (2)	−0.007 (2)	0.005 (2)	−0.001 (2)
C	0.084 (8)	0.044 (5)	0.052 (6)	−0.003 (6)	0.019 (6)	0.009 (5)
C1	0.007 (2)	0.017 (3)	0.016 (3)	0.003 (2)	0.001 (2)	0.000 (2)
C2	0.006 (2)	0.018 (3)	0.014 (3)	0.002 (2)	0.003 (2)	0.001 (2)
C3	0.011 (3)	0.019 (3)	0.024 (3)	0.007 (2)	0.003 (2)	0.005 (3)
C4	0.025 (3)	0.033 (4)	0.023 (3)	−0.015 (3)	0.012 (3)	−0.002 (3)
C5	0.024 (3)	0.024 (3)	0.014 (3)	0.005 (3)	0.013 (2)	0.002 (3)
C6	0.015 (3)	0.022 (3)	0.011 (3)	0.005 (2)	0.002 (2)	0.001 (2)
C7	0.046 (5)	0.042 (5)	0.024 (3)	−0.009 (4)	0.020 (3)	−0.012 (3)
C8	0.012 (3)	0.017 (3)	0.025 (3)	0.003 (2)	0.008 (2)	0.001 (3)
C9	0.013 (3)	0.016 (3)	0.011 (2)	0.006 (2)	0.000 (2)	−0.001 (2)
C10	0.019 (3)	0.020 (3)	0.027 (3)	−0.008 (3)	0.011 (3)	−0.008 (3)
C11	0.027 (4)	0.025 (4)	0.026 (3)	−0.016 (3)	0.007 (3)	−0.011 (3)
C12	0.011 (3)	0.023 (3)	0.019 (3)	−0.007 (2)	0.006 (2)	−0.001 (3)
C13	0.011 (3)	0.027 (3)	0.015 (3)	0.003 (2)	0.004 (2)	0.004 (3)
C14	0.015 (3)	0.033 (4)	0.022 (3)	0.006 (3)	0.006 (3)	0.004 (3)
C15	0.027 (4)	0.043 (4)	0.038 (4)	0.003 (3)	0.027 (3)	0.007 (3)
C16	0.014 (3)	0.029 (4)	0.031 (3)	0.008 (3)	0.014 (3)	0.000 (3)
C17	0.019 (3)	0.027 (3)	0.020 (3)	0.004 (3)	0.006 (2)	0.001 (3)
C18	0.029 (4)	0.038 (4)	0.052 (5)	0.000 (4)	0.011 (4)	−0.030 (4)
C19	0.012 (3)	0.019 (3)	0.024 (3)	0.005 (2)	0.006 (2)	−0.001 (3)
C20	0.009 (3)	0.026 (3)	0.015 (3)	0.002 (2)	0.001 (2)	0.001 (3)
C11A	0.012 (3)	0.026 (3)	0.017 (3)	−0.001 (2)	0.008 (2)	0.004 (3)
C12A	0.022 (3)	0.027 (4)	0.024 (3)	−0.002 (3)	−0.001 (3)	−0.004 (3)
C21A	0.017 (3)	0.028 (3)	0.019 (3)	0.006 (3)	0.008 (2)	−0.003 (3)
C22A	0.024 (3)	0.035 (4)	0.034 (4)	−0.004 (3)	0.011 (3)	0.007 (3)

### Geometric parameters (Å, °)

**Table:**

Co1—N2	1.861 (5)	C4—H4B	0.9800
Co1—N1	1.871 (5)	C4—H4C	0.9800
Co1—O2	1.887 (4)	C5—C6	1.391 (9)
Co1—O1	1.891 (4)	C5—H5A	0.9500
Co1—O21A	1.902 (5)	C6—C8	1.425 (9)
Co1—O11A	1.929 (4)	C7—H7A	0.9800
Co2—O12A^i^	2.043 (4)	C7—H7B	0.9800
Co2—O12A	2.043 (4)	C7—H7C	0.9800
Co2—O2^i^	2.117 (4)	C8—C9	1.451 (8)
Co2—O2	2.117 (4)	C9—H9A	0.9500
Co2—O1	2.160 (4)	C10—C11	1.511 (9)
Co2—O1^i^	2.160 (4)	C10—H10A	0.9900
Cl1—C	1.763 (10)	C10—H10B	0.9900
Cl2—C	1.771 (13)	C11—H11A	0.9900
O1—C1	1.310 (7)	C11—H11B	0.9900
O2—C20	1.334 (7)	C12—C13	1.432 (9)
O3—C3	1.361 (8)	C12—H12A	0.9500
O3—C4	1.441 (8)	C13—C14	1.411 (9)
O4—C6	1.342 (7)	C13—C20	1.418 (9)
O4—C7	1.440 (8)	C14—C16	1.385 (10)
O5—C14	1.380 (8)	C15—H15A	0.9800
O5—C15	1.431 (8)	C15—H15B	0.9800
O6—C17	1.353 (8)	C15—H15C	0.9800
O6—C18	1.432 (9)	C16—C17	1.393 (9)
O11A—C11A	1.275 (7)	C16—H16A	0.9500
O12A—C11A	1.256 (8)	C17—C19	1.397 (9)
O21A—C21A	1.293 (8)	C18—H18A	0.9800
O22A—C21A	1.226 (8)	C18—H18B	0.9800
N1—C9	1.282 (8)	C18—H18C	0.9800
N1—C10	1.465 (8)	C19—C20	1.400 (9)
N2—C12	1.281 (8)	C19—H19A	0.9500
N2—C11	1.485 (8)	C11A—C12A	1.491 (9)
C—H0A	0.9900	C12A—H12B	0.9800
C—H0B	0.9900	C12A—H12C	0.9800
C1—C2	1.411 (8)	C12A—H12D	0.9800
C1—C8	1.433 (8)	C21A—C22A	1.500 (10)
C2—C3	1.366 (9)	C22A—H22A	0.9800
C2—H2A	0.9500	C22A—H22B	0.9800
C3—C5	1.412 (9)	C22A—H22C	0.9800
C4—H4A	0.9800		
			
N2—Co1—N1	86.4 (2)	O4—C7—H7A	109.5
N2—Co1—O2	93.9 (2)	O4—C7—H7B	109.5
N1—Co1—O2	178.7 (2)	H7A—C7—H7B	109.5
N2—Co1—O1	176.1 (2)	O4—C7—H7C	109.5
N1—Co1—O1	96.1 (2)	H7A—C7—H7C	109.5
O2—Co1—O1	83.65 (18)	H7B—C7—H7C	109.5
N2—Co1—O21A	96.4 (2)	C6—C8—C1	118.0 (6)
N1—Co1—O21A	91.4 (2)	C6—C8—C9	118.4 (6)
O2—Co1—O21A	89.90 (19)	C1—C8—C9	123.5 (6)
O1—Co1—O21A	86.66 (19)	N1—C9—C8	125.2 (6)
N2—Co1—O11A	86.1 (2)	N1—C9—H9A	117.4
N1—Co1—O11A	86.2 (2)	C8—C9—H9A	117.4
O2—Co1—O11A	92.57 (18)	N1—C10—C11	108.8 (5)
O1—Co1—O11A	90.98 (18)	N1—C10—H10A	109.9
O21A—Co1—O11A	176.38 (19)	C11—C10—H10A	109.9
O12A^i^—Co2—O12A	180.000 (1)	N1—C10—H10B	109.9
O12A^i^—Co2—O2^i^	86.78 (17)	C11—C10—H10B	109.9
O12A—Co2—O2^i^	93.22 (17)	H10A—C10—H10B	108.3
O12A^i^—Co2—O2	93.22 (17)	N2—C11—C10	108.0 (5)
O12A—Co2—O2	86.78 (17)	N2—C11—H11A	110.1
O2^i^—Co2—O2	180.0	C10—C11—H11A	110.1
O12A^i^—Co2—O1	92.92 (16)	N2—C11—H11B	110.1
O12A—Co2—O1	87.08 (16)	C10—C11—H11B	110.1
O2^i^—Co2—O1	107.85 (15)	H11A—C11—H11B	108.4
O2—Co2—O1	72.15 (15)	N2—C12—C13	124.7 (6)
O12A^i^—Co2—O1^i^	87.08 (16)	N2—C12—H12A	117.7
O12A—Co2—O1^i^	92.92 (16)	C13—C12—H12A	117.7
O2^i^—Co2—O1^i^	72.15 (15)	C14—C13—C20	117.5 (6)
O2—Co2—O1^i^	107.85 (15)	C14—C13—C12	119.5 (6)
O1—Co2—O1^i^	180.000 (1)	C20—C13—C12	123.0 (5)
C1—O1—Co1	125.3 (4)	O5—C14—C16	122.7 (6)
C1—O1—Co2	136.1 (4)	O5—C14—C13	114.7 (6)
Co1—O1—Co2	98.66 (17)	C16—C14—C13	122.6 (6)
C20—O2—Co1	122.9 (4)	O5—C15—H15A	109.5
C20—O2—Co2	135.6 (4)	O5—C15—H15B	109.5
Co1—O2—Co2	100.29 (18)	H15A—C15—H15B	109.5
C3—O3—C4	117.0 (5)	O5—C15—H15C	109.5
C6—O4—C7	117.7 (5)	H15A—C15—H15C	109.5
C14—O5—C15	116.9 (6)	H15B—C15—H15C	109.5
C17—O6—C18	118.9 (5)	C14—C16—C17	118.1 (6)
C11A—O11A—Co1	128.5 (4)	C14—C16—H16A	121.0
C11A—O12A—Co2	128.5 (4)	C17—C16—H16A	121.0
C21A—O21A—Co1	128.8 (4)	O6—C17—C16	115.1 (6)
C9—N1—C10	120.3 (5)	O6—C17—C19	122.8 (6)
C9—N1—Co1	125.0 (4)	C16—C17—C19	122.2 (6)
C10—N1—Co1	114.7 (4)	O6—C18—H18A	109.5
C12—N2—C11	122.4 (5)	O6—C18—H18B	109.5
C12—N2—Co1	125.5 (4)	H18A—C18—H18B	109.5
C11—N2—Co1	111.9 (4)	O6—C18—H18C	109.5
Cl1—C—Cl2	110.9 (7)	H18A—C18—H18C	109.5
Cl1—C—H0A	109.5	H18B—C18—H18C	109.5
Cl2—C—H0A	109.5	C17—C19—C20	118.8 (6)
Cl1—C—H0B	109.5	C17—C19—H19A	120.6
Cl2—C—H0B	109.5	C20—C19—H19A	120.6
H0A—C—H0B	108.0	O2—C20—C19	117.8 (5)
O1—C1—C2	118.2 (5)	O2—C20—C13	121.3 (6)
O1—C1—C8	122.0 (5)	C19—C20—C13	120.9 (6)
C2—C1—C8	119.8 (5)	O12A—C11A—O11A	126.6 (6)
C3—C2—C1	119.9 (6)	O12A—C11A—C12A	118.5 (5)
C3—C2—H2A	120.0	O11A—C11A—C12A	114.9 (6)
C1—C2—H2A	120.0	C11A—C12A—H12B	109.5
O3—C3—C2	124.1 (6)	C11A—C12A—H12C	109.5
O3—C3—C5	113.6 (6)	H12B—C12A—H12C	109.5
C2—C3—C5	122.3 (6)	C11A—C12A—H12D	109.5
O3—C4—H4A	109.5	H12B—C12A—H12D	109.5
O3—C4—H4B	109.5	H12C—C12A—H12D	109.5
H4A—C4—H4B	109.5	O22A—C21A—O21A	127.5 (6)
O3—C4—H4C	109.5	O22A—C21A—C22A	119.8 (6)
H4A—C4—H4C	109.5	O21A—C21A—C22A	112.8 (6)
H4B—C4—H4C	109.5	C21A—C22A—H22A	109.5
C6—C5—C3	118.5 (5)	C21A—C22A—H22B	109.5
C6—C5—H5A	120.8	H22A—C22A—H22B	109.5
C3—C5—H5A	120.8	C21A—C22A—H22C	109.5
O4—C6—C5	122.9 (5)	H22A—C22A—H22C	109.5
O4—C6—C8	115.8 (5)	H22B—C22A—H22C	109.5
C5—C6—C8	121.4 (6)		
			
N1—Co1—O1—C1	18.7 (5)	Co2—O1—C1—C8	160.4 (4)
O2—Co1—O1—C1	−162.6 (5)	O1—C1—C2—C3	175.2 (5)
O21A—Co1—O1—C1	−72.3 (5)	C8—C1—C2—C3	−3.6 (8)
O11A—Co1—O1—C1	104.9 (5)	C4—O3—C3—C2	1.8 (9)
N1—Co1—O1—Co2	−160.9 (2)	C4—O3—C3—C5	179.5 (5)
O2—Co1—O1—Co2	17.86 (18)	C1—C2—C3—O3	178.4 (5)
O21A—Co1—O1—Co2	108.13 (19)	C1—C2—C3—C5	0.9 (9)
O11A—Co1—O1—Co2	−74.62 (18)	O3—C3—C5—C6	−176.8 (5)
O12A^i^—Co2—O1—C1	71.5 (5)	C2—C3—C5—C6	1.0 (9)
O12A—Co2—O1—C1	−108.5 (5)	C7—O4—C6—C5	4.5 (9)
O2^i^—Co2—O1—C1	−16.1 (6)	C7—O4—C6—C8	−175.5 (6)
O2—Co2—O1—C1	163.9 (6)	C3—C5—C6—O4	180.0 (5)
O12A^i^—Co2—O1—Co1	−109.03 (19)	C3—C5—C6—C8	−0.1 (9)
O12A—Co2—O1—Co1	70.97 (19)	O4—C6—C8—C1	177.4 (5)
O2^i^—Co2—O1—Co1	163.42 (17)	C5—C6—C8—C1	−2.5 (9)
O2—Co2—O1—Co1	−16.58 (17)	O4—C6—C8—C9	−1.7 (8)
N2—Co1—O2—C20	−32.5 (5)	C5—C6—C8—C9	178.4 (6)
O1—Co1—O2—C20	150.6 (5)	O1—C1—C8—C6	−174.4 (5)
O21A—Co1—O2—C20	63.9 (5)	C2—C1—C8—C6	4.4 (8)
O11A—Co1—O2—C20	−118.7 (5)	O1—C1—C8—C9	4.6 (9)
N2—Co1—O2—Co2	158.6 (2)	C2—C1—C8—C9	−176.6 (5)
O1—Co1—O2—Co2	−18.32 (19)	C10—N1—C9—C8	175.5 (6)
O21A—Co1—O2—Co2	−105.0 (2)	Co1—N1—C9—C8	−2.7 (8)
O11A—Co1—O2—Co2	72.4 (2)	C6—C8—C9—N1	−173.9 (6)
O12A^i^—Co2—O2—C20	−57.9 (6)	C1—C8—C9—N1	7.1 (9)
O12A—Co2—O2—C20	122.1 (6)	C9—N1—C10—C11	166.1 (6)
O1—Co2—O2—C20	−149.9 (6)	Co1—N1—C10—C11	−15.5 (7)
O1^i^—Co2—O2—C20	30.1 (6)	C12—N2—C11—C10	149.8 (6)
O12A^i^—Co2—O2—Co1	108.7 (2)	Co1—N2—C11—C10	−34.2 (6)
O12A—Co2—O2—Co1	−71.3 (2)	N1—C10—C11—N2	30.8 (7)
O1—Co2—O2—Co1	16.70 (17)	C11—N2—C12—C13	174.9 (6)
O1^i^—Co2—O2—Co1	−163.30 (17)	Co1—N2—C12—C13	−0.6 (9)
N2—Co1—O11A—C11A	−133.8 (5)	N2—C12—C13—C14	170.6 (6)
N1—Co1—O11A—C11A	139.6 (5)	N2—C12—C13—C20	−12.1 (10)
O2—Co1—O11A—C11A	−40.1 (5)	C15—O5—C14—C16	−0.3 (9)
O1—Co1—O11A—C11A	43.6 (5)	C15—O5—C14—C13	179.3 (6)
O2^i^—Co2—O12A—C11A	−140.5 (5)	C20—C13—C14—O5	−177.6 (5)
O2—Co2—O12A—C11A	39.5 (5)	C12—C13—C14—O5	−0.2 (9)
O1—Co2—O12A—C11A	−32.8 (5)	C20—C13—C14—C16	2.0 (10)
O1^i^—Co2—O12A—C11A	147.2 (5)	C12—C13—C14—C16	179.4 (6)
N2—Co1—O21A—C21A	−37.0 (6)	O5—C14—C16—C17	179.8 (6)
N1—Co1—O21A—C21A	49.5 (5)	C13—C14—C16—C17	0.2 (10)
O2—Co1—O21A—C21A	−130.8 (5)	C18—O6—C17—C16	173.6 (6)
O1—Co1—O21A—C21A	145.5 (5)	C18—O6—C17—C19	−6.2 (10)
N2—Co1—N1—C9	175.4 (5)	C14—C16—C17—O6	179.2 (6)
O1—Co1—N1—C9	−7.7 (5)	C14—C16—C17—C19	−0.9 (10)
O21A—Co1—N1—C9	79.1 (5)	O6—C17—C19—C20	179.1 (6)
O11A—Co1—N1—C9	−98.3 (5)	C16—C17—C19—C20	−0.8 (10)
N2—Co1—N1—C10	−2.9 (4)	Co1—O2—C20—C19	−153.3 (4)
O1—Co1—N1—C10	174.0 (4)	Co2—O2—C20—C19	11.0 (9)
O21A—Co1—N1—C10	−99.2 (4)	Co1—O2—C20—C13	29.1 (8)
O11A—Co1—N1—C10	83.4 (4)	Co2—O2—C20—C13	−166.6 (4)
N1—Co1—N2—C12	−162.8 (6)	C17—C19—C20—O2	−174.5 (6)
O2—Co1—N2—C12	18.5 (5)	C17—C19—C20—C13	3.1 (9)
O1—Co1—N2—C12	69 (3)	C14—C13—C20—O2	173.8 (6)
O21A—Co1—N2—C12	−71.9 (5)	C12—C13—C20—O2	−3.5 (9)
O11A—Co1—N2—C12	110.8 (5)	C14—C13—C20—C19	−3.6 (9)
N1—Co1—N2—C11	21.3 (4)	C12—C13—C20—C19	179.0 (6)
O2—Co1—N2—C11	−157.4 (4)	Co2—O12A—C11A—O11A	−4.4 (9)
O21A—Co1—N2—C11	112.3 (4)	Co2—O12A—C11A—C12A	175.4 (4)
O11A—Co1—N2—C11	−65.1 (4)	Co1—O11A—C11A—O12A	1.6 (9)
Co1—O1—C1—C2	162.2 (4)	Co1—O11A—C11A—C12A	−178.2 (4)
Co2—O1—C1—C2	−18.5 (8)	Co1—O21A—C21A—O22A	11.6 (10)
Co1—O1—C1—C8	−19.0 (8)	Co1—O21A—C21A—C22A	−167.3 (4)

Symmetry codes: (i) −*x*+1, −*y*+1, −*z*+1.

### Hydrogen-bond geometry (Å, °)

**Table:**

*D*—H···*A*	*D*—H	H···*A*	*D*···*A*	*D*—H···*A*
C—H0A···O22A	0.99	2.33	3.269 (13)	158
C4—H4A···O6^ii^	0.98	2.35	3.326 (8)	175
C7—H7A···O6^iii^	0.98	2.51	3.421 (9)	156
C11—H11A···O3^iii^	0.99	2.62	3.602 (8)	174
C11—H11B···Cl1^iv^	0.99	2.73	3.664 (8)	158
C15—H15A···O4^v^	0.98	2.64	3.568 (10)	158
C12A—H12B···Cl1^iii^	0.98	2.91	3.354 (8)	108

Symmetry codes: (ii) *x*+1/2, −*y*+3/2, *z*−1/2; (iii) −*x*+1/2, *y*−1/2, −*z*+1/2; (iv) *x*+1/2, −*y*+1/2, *z*+1/2; (v) *x*−1/2, −*y*+1/2, *z*+1/2.
